# Variation in Bioactive Compounds and Antioxidant Activity of *Rubus* Fruits at Different Developmental Stages

**DOI:** 10.3390/foods11081169

**Published:** 2022-04-18

**Authors:** Xin Huang, Yaqiong Wu, Shanshan Zhang, Hao Yang, Wenlong Wu, Lianfei Lyu, Weilin Li

**Affiliations:** 1 Jiangsu Key Laboratory for the Research and Utilization of Plant Resources, Institute of Botany, Jiangsu Province and Chinese Academy of Sciences, The Jiangsu Provincial Platform for Conservation and Utilization of Agricultural Germplasm, Qian Hu Hou Cun No. 1, Nanjing 210014, China; huangx19980601@163.com (X.H.); 15943097183@163.com (S.Z.); 1964wwl@163.com (W.W.); njbglq@163.com (L.L.); 2 Co-Innovation Center for Sustainable Forestry in Southern China, Nanjing Forestry University, 159 Longpan Road, Nanjing 210037, China; yanghao_19940720@163.com

**Keywords:** blackberry, raspberry, antioxidant activity, active substances

## Abstract

Blackberry and raspberry have high nutritional, health value, and are popular with consumers for their unique flavors. To explore the relationships between nutrient accumulation, antioxidant substance contents in blackberry and raspberry fruits, and fruit growth and development, seven *Rubus* cultivars were selected, and contents of the main active substance were determined. “Clode Summit” had the highest soluble sugar and fructose contents, “Chester”—the highest total phenol content, and “Bristol’—the highest anthocyanin content. Generally, the contents of flavonoids and total phenols showed a downward trend with the development of fruit in seven *Rubus* cultivars, and the content of anthocyanins increased rapidly in the later stage of development. Pearson correlation analysis showed extremely significant correlation between antioxidant activity and the contents of vitamin E, total phenols, and flavonoids. Flavonoids were extremely significantly positively correlated with the content of total phenols, and the contents of flavonoids and anthocyanins in various cultivars were highly negatively correlated. Considering the different nutritional ingredients and active antioxidant substance contents, “Clode Summit”, “Bristol”, and “Chester” are recommended for raw consumption, processing, and medicinal purposes, respectively. These results provide a reference for comparing the main active substance contents in different *Rubus* cultivars and their changes across fruit development stages.

## 1. Introduction

Blackberry and raspberry, which belong to the genus *Rubus* in the family Rosaceae, are small perennial berry fruit trees. Berries are one of third-generation fruits that are popular in Europe. These fruits are rich in sugars, fat, vitamin C, vitamin E, ellagic acid, flavones, anthocyanins, etc. Moreover, these berries have antibacterial, anti-inflammatory, antioxidant, antiaging, and other effects [1,2,3,4,5] and are favored by many consumers on the market. The main difference between blackberry and raspberry is that the aggregate fruits of blackberry do not separate from the receptacle when ripe, while raspberry fruits separate from the receptacle. Depending on the color and growth characteristics of the fruit, raspberry can be divided into four types: red raspberry, yellow raspberry, black raspberry, and purple raspberry. Purple raspberry is a hybrid between black raspberry and red raspberry with growth characteristics similar to those of black raspberry [6].

Blackberry and raspberry are highly adaptable to environmental conditions such as moisture, climate, and soil and have the advantages of drought resistance, resistance to diseases and insect pests, vigorous growth, and easy reproduction. Blackberry is native to North America and has a long history of cultivation in Europe and America. In 1986, blackberry was first introduced to China by the Institute of Botany, Chinese Academy of Sciences, Jiangsu Province, and later promoted. Based on the investigation and collection of domesticated and wild plant germplasm resources of *Rubus*, the selection and breeding of blackberry have been carried out [7]. The cultivation of raspberry originated in Europe and has a history of more than 100 years. China only began to grow raspberry on a large scale in the 1980s, and most of the plants were introduced from abroad [8]. Blackberries and raspberries have high economic importance in several countries, including America, Canada, and China [7,9]. Because of its high nutritional value and antioxidant effect, these fruits have good market and commercial value, both at the national and international levels [8,10]. At present, the complete chloroplast genome sequence of high-quality hybrid blackberry has been obtained [11], and the extraction process of blackberry phenols has also been optimized and improved [12]. Research on the nutrient composition and content of active antioxidant substances of mature fruits of some blackberry cultivars found that blackberry fruits contain 15 important phenolic substances, and cyanidin-3-*O*-glucoside (CG) and chlorogenic acid (CA) are responsible for the antioxidant activity of blackberry. The main free antioxidant component, ferulic acid (FA), is the main binding component leading to the antioxidant capacity of blackberry [13]. Moreover, related studies have also found that a large amount of active phenolic substances can be extracted from raspberry leaves [14]. The anthocyanin content in mature blackberry fruit is significantly higher than that in red raspberry [15]. However, there is currently a lack of systematic research on the changes in the nutrient substance contents and antioxidant ability of blackberry, raspberry, and blackberry–raspberry hybrids at different developmental stages, as well as the difference in the contents of the main active substances among different cultivars.

Thus, two blackberry cultivars “Chester” and “Hull”, two blackberry–raspberry hybrids “Boysen” and “Young”, and three raspberry cultivars with different fruit colors, “Bristol” (black raspberry), “Heritage” (red raspberry), and “Clode Summit” (yellow raspberry), were selected to study the sugar, vitamin C, vitamin E, total phenol, anthocyanin and flavonoid contents, and the antioxidant capacity of the fruits during different growth stages. Additionally, the relationship between the changes in these nutritional indicators and fruit development was analyzed to provide a reference for the selection of cultivars/periods with high active substance contents in their fruits and to gain an in-depth understanding of how fruit inclusions accumulate in representative cultivars of *Rubus*.

## 2. Materials and Methods

### 2.1. Plant Materials

**Figure 1:**
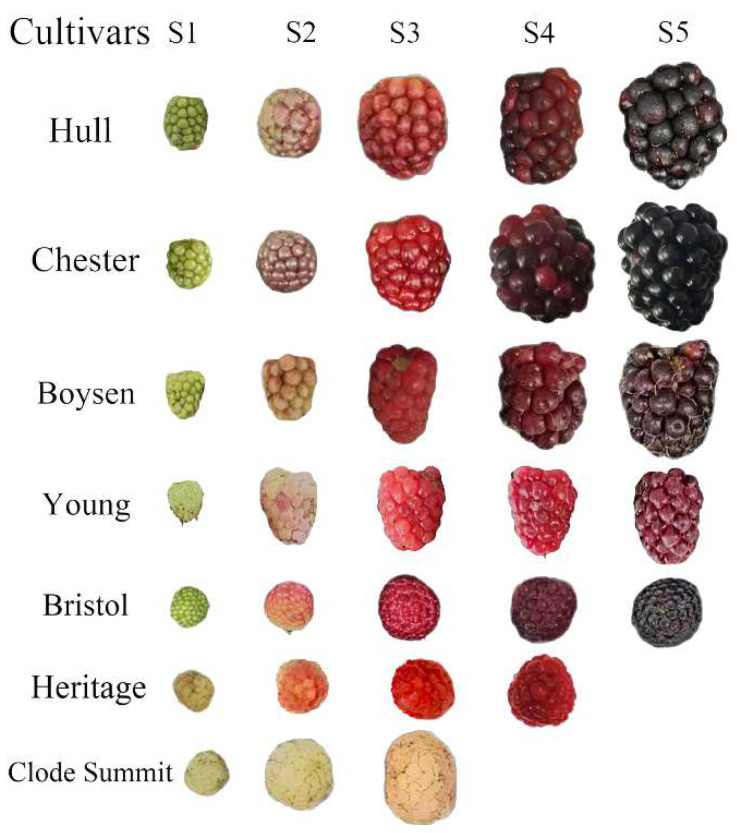
Appearance of different cultivars of *Rubus* in different fruit developmental stages.

Seven cultivars including blackberry cultivars “Chester” and “Hull”, raspberry cultivars “Bristol”, “Heritage”, and “Clode Summit”, and blackberry–raspberry hybrids “Boysen” and “Young” were representatively selected as test materials and were obtained from the Baima Test Base in Lishui District, Nanjing, Jiangsu Province. The base belongs to the subtropical monsoon climate zone. The specific location is 119°11′ E, 31°36′ N. The living conditions were as follows: annual average temperature 15.6 °C, annual rainfall 1031.9 mm, average sunshine 50%, average temperature from June to August 27.1 °C, rainfall 435.6 mm, and soil pH 5.52 with the following composition: organic matter 18.67 g·kg^−1^, total nitrogen 1.25 g·kg^−1^, available phosphorus 4.83 mg·kg^−1^, and available potassium 94.21 mg·kg^−1^. The sampling period was distinguished according to the color of the fruit. For “Chester”, “Hull”, and “Bristol”, green fruits, green–red fruits, red fruits, red–purple fruits, and purple fruits were picked; for red raspberry cultivars, green fruits, green–red fruits, red fruits, and dark red fruits were picked; and for yellow raspberry cultivars, green fruits, multicolor fruits, and yellow fruits were picked (Figure 1). Three biological replicates were sampled, and fruits from the east, west, south, and north sides of plants were collected for each biological sample and mixed.

### 2.2. Measurement of Fruit Horizontal Diameter, Vertical Diameter, Weight, and Hardness

At least 6 fruits were selected for sampling at each time point. Their horizontal and vertical diameters were measured with a Vernier caliper, and each fruit was weighed with a balance with an accuracy of 0.01 g. The hardness of the fruits that developed to the last two stages was determined with a Japan Takemura Cat No. 9300 (km^−5^) fruit hardness tester. The diameter of the probe was 5 mm, the pressing distance was 10 mm, and the maximum breaking force was used as the hardness. The values are expressed as kg·cm^−2^. Each test was repeated 6 times, and the average value was obtained.

### 2.3. Determination of Fruit Sugar and Protein Content

Soluble sugar content (SSC) was determined by the anthrone colorimetric method [16]. A total of 0.2 g of fruit was weighed, added to 2 mL of distilled water, ground into a slurry with a homogenizer, boiled in a water bath for 10 min, and then centrifuged at 4000 rpm for 10 min after cooling. Anthrone and sulfuric acid were added, and the mixture was boiled in a water bath for 10 min. The absorbance value was measured at 630 nm. Glucose content (GC) was determined by the glucose oxidase method [17]. Moreover, fructose content (FC) was measured using a fructose quantitative detection kit (Nanjing Jiancheng Institute of Bioengineering, Nanjing, China) which include a reaction solution and a fructose standard. Fresh fruit (1 g) was added to 10 mL of water, homogenized, and centrifuged at 3500 rpm for 10 min. The supernatant was diluted to a suitable concentration, added to the matrix reaction solution, reacted accurately in a boiling water bath for 8 min, and cooled under running water. The product generated by the reaction of fructose with the matrix solution had an absorption peak at 285 nm, so the optical density (OD) was measured at 285 nm by UV spectrophotometry.

$$FC=\frac{A_{2}}{A_{1}}\times C\times n$$

where *A*_1_ is the absorbance of the standard, *A*_2_ is the absorbance of the test sample, *C* is the concentration of the standard, and *n* is the dilution factor.

The Coomassie brilliant blue method [18] was used to determine the protein content (PC). One gram of fruit was weighed, and reagents were added to achieve a weight (g)/volume (mL) ratio of 1:9; then, the sample was homogenized and centrifuged at 4000× *g* for 10 min. The supernatant and Coomassie G-250 were combined and incubated at room temperature for 5 min. The absorbance was measured at 595 nm. The protein content was calculated, and the protein content of the samples was expressed as milligram per gram of FW (mg/g FW).

### 2.4. Determination of Antioxidant Capacity

The free radical scavenging capacity of 2′-diphenyl-1-picrylhydrazyl (DPPH) was measured according to the method of the study of Xie et al. [19], with slight modifications. A total of 0.2 g of fruit was weighed, added to 1 mL of 80% methanol solution, paced in an ice-water bath, homogenized, and centrifuged at 12,000× *g* for 10 min. The supernatant was collected and placed on ice for testing. Each sample included a control and measurement tubes. A total of 400 µL of sample supernatant was added to each tube, 600 of µL 80% methanol was added to the control tube, 600 µL of working solution was added to the measurement tube, and 400 µL of 80% methanol and 600 µL of working solution were added to the blank tube. The solutions were mixed well and allowed to stand for 30 min at 25 °C in the dark. The tubes were then centrifuged at 4000 rpm for 5 min. The absorbance was measured at 517 nm. Trolox was used as a positive control. Six Trolox concentrations were analyzed in triplicate. The sample concentration at 50% inhibition of DPPH radicals (IC50) was calculated by fitting the percent inhibition of six concentrations (0.25–2.5 mg/mL). The free radical scavenging rate (*FRSR*) was calculated according to the following formula.

$$FRSR=(1-\frac{\left(A_{2}-A_{1}\right)}{A_{0}})\times 100\%\;IC_{50}=C\times \frac{V}{m}\times n$$

where *A*_0_ is the absorbance of double-distilled water, *A*_1_ is the absorbance of the standard, *A*_2_ is the absorbance of the test sample, *V* is the volume of the extract solution (in milliliters), *n* is the dilution factor, *m* is the weight of the sample (in grams), and *C* is the concentration equivalent to Trolox substituted into the standard curve. The equation obtained for the calibration curve of the standard solution in the range of 0–30 µg/mL was y = 0.032x + 0.0094 (R^2^ = 0.9981).

### 2.5. Determination of Vitamin Contents

Commercial kits (Nanjing Jiancheng Institutes of Biological Engineering) were used for the determination of vitamin E content (VEC) and vitamin C content (VCC). VE and VC catalyze Fe^3+^ to form Fe^2+^ and then form a pink complex with phenanthroline. To extract VE and VC, the reagents were added to achieve a weight (g)/volume (mL) ratio of 1:9. The mixture was homogenized under ice-water bath conditions and centrifuged to transfer the supernatant to a centrifuge tube. Further extraction of VE required absolute ethanol and n-heptane to be added in sequence, and the solution was then vortexed and centrifuged. The reagents were added according to the requirements of the kit and mixed well, and the absorbance was measured at 533 and 536 nm separately.

### 2.6. Determination of the Flavonoid Content

To estimate the flavonoid content in the Rubus, the calorimetric method using aluminum chloride is widely applied [20]. Flavonoids and Al^3+^ form red complexes in alkaline nitrite solution, and there is an absorption peak at 502 nm. The total flavonoid content (TFC) in the sample can be calculated by measuring the absorbance value. Samples were washed with normal saline, dried, then ground with liquid nitrogen to a powder, weighed to 0.05 g and combined with 2 mL of 60% ethanol. The solution was shaken and extracted at 60 °C for 2 h and centrifuged at 10,000 rpm for 10 min at room temperature. The supernatant was collected for testing. According to the standard curve generation procedure, each sample was tested in triplicate, and the average value was taken and substituted into the standard curve to calculate the flavonoid content. The equation obtained for the calibration curve of the rutin standard solution in the range of 0–0.1 mg/mL was y = 9.0771x + 0.0141 (R^2^ = 0.9998). The flavonoid content of the samples was expressed as milligam per gram of FW (mg/g FW).

### 2.7. Determination of the Anthocyanin Content

Total anthocyanin content (TAC) was measured by the pH difference method [21], with slight modifications. Three grams of fruit homogenate was added to 30 mL of 50% ethanol, shaken well, and sonicated at 35 °C and 60 Hz for 20 min. The mixture was centrifuged at 5000 rpm for 5 min, and 0.5 mL of the supernatant was transferred to a 10 mL centrifuge tube, added to 4.5 mL of pH 1.0 buffer solution, reacted at room temperature for 20 min, and adjusted to an absorbance of zero with double-distilled water, and the absorbance was measured at 510 nm. A standard curve was utilized to calculate the TAC. The equation obtained for the calibration curve of the cyanidin-3-glucoside standard solution in the range of 0–0.6 mg/mL was y = 0.0455x − 0.0881 (R^2^ = 0.9996). The results are expressed as milligram per gram of FW (mg/g FW).

### 2.8. Determination of the Total Phenol Content

The concentration of the total phenolic compounds was determined according to Waterhouse’s method [22]. Phenol and tungstomolybdic acid form blue compounds under alkaline conditions, and there is an absorption peak at 760 nm. The total phenol content (TPC) in the sample can be calculated by measuring the absorbance value. The fresh fruits were ground into powder with liquid nitrogen, weighed to 0.1 g, added to 2 mL of extract solution (60% aqueous ethanol), and mixed for 3 min, sonicated at 60 °C for 30 min, and then centrifuged at 4000 rpm for 10 min. The supernatant was transferred to a centrifuge tube, with reagents added as required, mixed, and allowed to stand at room temperature for 10 min. The absorbance of each tube was measured at a wavelength of 760 nm, The hydromethanolic gallic acid solution was freshly prepared in a series of concentrations (0.1–1 mM) and tested in parallel to establish the calibration curve. The total phenolic content of the samples was calculated as milligram of gallic acid equivalent per gram of fresh sample (mg/g FW).

### 2.9. Data Processing

The data are expressed as mean ± standard deviation (SD), and all experiments were performed using the SPSS 24.0 statistical software program (SPSS, Chicago, IL, USA). Using single-factor ANOVA to compare the averages, Duncan’s post-multiple comparison was utilized to calculate significant differences between the transverse diameter, longitudinal diameter, quality, SSC, GC, FC, VCC, VEC, TPC, TFC, TAC, and DPPH free radical scavenging capacity (*p* < 0.05). Pearson correlation analysis was used to evaluate the relationship between the SSC, GC, FC, VCC, VEC, TPC, TFC, TAC, and DPPH free radical scavenging capacity.

## 3. Results

### 3.1. Fruit Morphological Index Analysis

In this experiment, the results showed that the weight of mature blackberry fruits was significantly higher than that of raspberry fruits. The average weight of mature “Chester” fruits was the largest, reaching 6.84 g, while mature “Heritage” fruits weighed only 1.46 g (Table 1). Hybrids were second only to blackberry in terms of fruit size and weight. Through the analysis of the horizontal and vertical diameters of the fruits of the seven cultivars at different stages, it was concluded that blackberry had the fastest growth and development during the period from green–red fruit to red fruit, and there was a certain amount of growth during each period. The fruit weight of the hybrid cultivars remained unchanged from the red to red–purple development stage. The growth performance of different raspberry types varied. Black raspberry grew rapidly in the initial stage, developed slowly from green–red to red–purple, and then rapidly developed. Red raspberry continued their initial growth rate, while the volume and quality decreased slightly in the ripe fruit; yellow raspberry continued to grow steadily over time. Moreover, we tested fruit hardness in the last two stages of fruit development. All cultivars had a significant decrease in fruit hardness, and the hardness of fully mature fruits was relatively low.

### 3.2. Fruit Sugar and Protein Content Analysis

**Figure 2:**
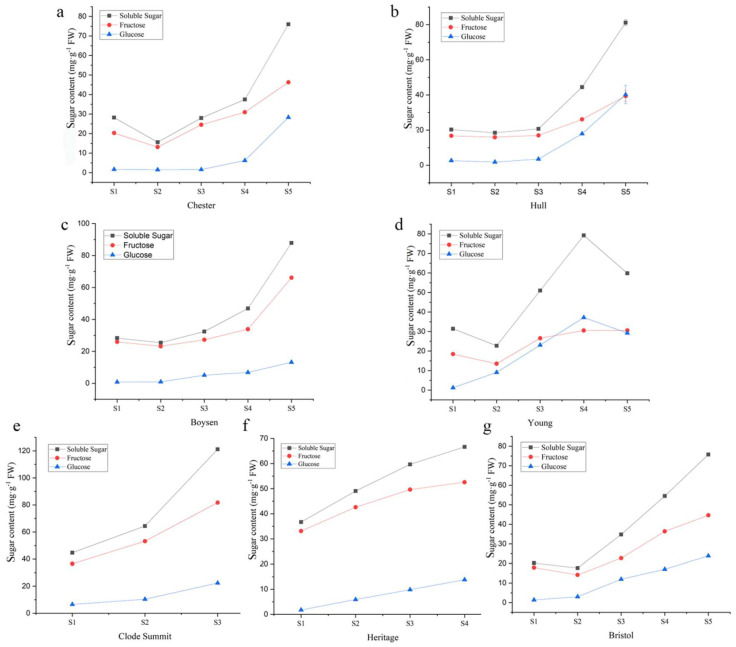
Variation in the sugar content in fruits of different *Rubus* cultivars at different developmental stages. (**a**) Chester; (**b**) Hull; (**c**) Boysen; (**d**) Young; (**e**) Clode Summit; (**f**) Heritage; and (**g**) Bristol. Black dashed lines represent soluble sugars, red dashed lines represent fructose, and blue dashed lines represent glucose.

The SSC, GC, and FC in fruits of seven Rubus cultivars at different developmental stages were systematically analyzed. The results showed that the SSC of “Chester” (Figure 2a), “Hull” (Figure 2b), “Boysen” (Figure 2c), and “Bristol” (Figure 2g) first decreased and then rapidly increased throughout the fruit development stage; the SSC of “Clode Summit” (Figure 2e) and “Heritage” (Figure 2f) continued to increase, and the change in SSC in “Young” first decreased, then increased and finally decreased again (Figure 2d). By comparing the SSC in mature fruits, it was found that the SSC of “Clode Summit” fruits was significantly higher than that of other fruits, reaching 121.16 mg·g^−1^ FW, and the content in “Young” fruits was the lowest at 59.85 mg·g^−1^ FW. The changes in GC were similar to those in SSC. The GC of “Clode Summit” (Figure 2e) fruits increased sharply in the last stage of fruit development, that of “Bristol” (Figure 2g) and “Heritage” (Figure 2f) fruits continued to increase, and that of “Young” (Figure 2d) fruits first increased steadily and then decreased. The GC in mature “Hull” fruits was the highest, reaching 40.22 mg·g^−1^ FW, and that in “Boysen” fruits was the lowest, at 13.14 mg·g^−1^ FW (Figure 2c). Interestingly, the change in FC was slightly different from that in SSC. The FC in “Chester” (Figure 2a), “Hull” (Figure 2b) “Boysen” (Figure 2c), and “Young” (Figure 2d) fruits first decreased and then increased, that in “Bristol” fruits first increased and then decreased (Figure 2g), that in “Clode Summit” fruits also continued to increase (Figure 2e), and that in “Heritage” fruits first increased and then remained unchanged (Figure 2f). The FC in “Clode Summit” fruits was the highest at 81.74 mg·g^−1^ FW, and in “Young” fruits, it was the lowest at 30.57 mg·g^−1^ FW. Moreover, the protein content in the fruits of each cultivar and period was not high. The relative content of protein in the green fruit period was the highest, except for “Clode Summit”, which had the highest protein content in the mature fruit (Supplementary Table S1).

### 3.3. Active Antioxidant Substance Content Analysis

**Figure 3:**
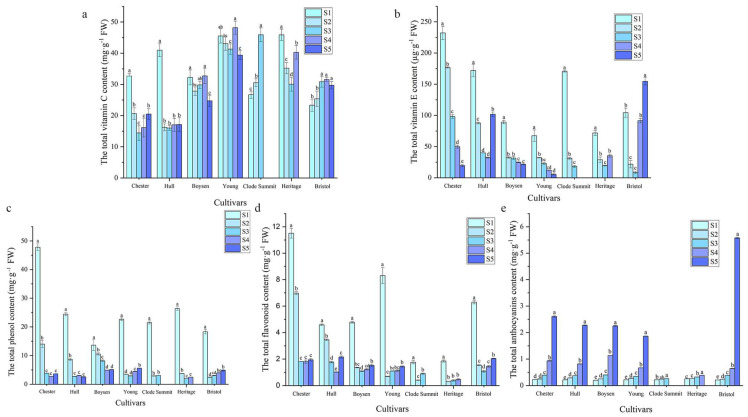
Variation in active antioxidant substance content in fruits of different *Rubus* cultivars at different developmental stages. (**a**) Vitamin C; (**b**) vitamin E; (**c**) total phenols; (**d**) total flavonoids; and (**e**) anthocyanins. Different letters indicate significant differences between the populations (*p* < 0.05). Different colors represent different harvest periods.

Plant fruits contain a variety of active antioxidant substances [23,24]. We analyzed the main biologically active substances in Rubus fruits, including the VCC, VEC, TPC, TFC, and TAC. The VCC in “Young”, “Boysen”, “Heritage”, and “Bristol” fruits changed slightly (Figure 3a). “Chester” and “Hull” fruits contained more vitamin C during the green fruit stage than in later stages, and the content gradually decreased as the fruits grew and developed; in contrast, “Clode Summit” fruits had a lower VCC during the green fruit stage, and the content gradually increased as the fruits grew and developed. Among mature fruits, “Heritage” fruits had the highest VCC, which was 48.81 mg·g^−1^ FW. The VEC of “Chester”, “Young”, and “Clode Summit” fruits was the highest during the fruiting period and then gradually decreased, while the VEC of “Hull”, “Boysen”, “Heritage”, and “Bristol” fruits first decreased and then increased (Figure 3b). Green fruits contained the highest amount of vitamin E, among which “Chester” fruits exhibited the highest VEC, reaching 103.2 mg·g^−1^ FW. The TPC of the seven cultivars showed a sharp decline in the early stage and a gentle trend in the later period (Figure 3c). The TPC in the green fruit period was significantly higher than that in other periods. The TPC in the “Chester” green fruit period was the highest, reaching 40.68 mg·g^−1^ FW. The change in TFC was similar to that in TPC. The TFC was the highest during the green fruit stage and then decreased sharply, but it increased slightly in the later stage of fruit development (Figure 3d). The TFC was still the highest in the “Chester” green fruit stage, reaching 11.24 mg·g^−1^ FW, and in “Clode Summit” and “Heritage” fruits, it was relatively low, 1.76 mg·g^−1^ FW and 1.90 mg·g^−1^ FW, respectively. The TAC of “Chester”, “Hull”, “Young”, “Boysen”, and “Bristol” fruits increased significantly from the red–purple fruit period to the purple fruit period (Figure 3e), indicating that anthocyanins were mainly synthesized and accumulated in the later stages of fruit development. The TAC of “Clode Summit” and “Heritage” fruits was significantly lower than that of other cultivars, especially “Clode Summit”. The highest TAC was observed in the mature fruits of the “Bristol” cultivar, reaching 5.59 mg·g^−1^ FW.

### 3.4. Antioxidant Ability Analysis

**Figure 4:**
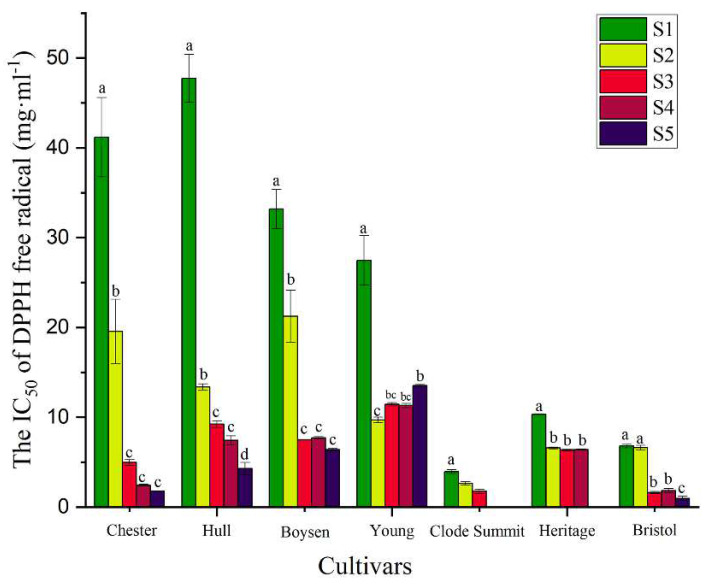
Variation in antioxidant activities. Different letters indicate significant differences between the populations (*p* < 0.05). Different colors represent different periods of harvest.

We used the DPPH free radical scavenging capacity to evaluate the antioxidant ability of the fruit of seven Rubus cultivars at different developmental stages. The results showed that the antioxidant ability of fruits at different developmental stages and between different cultivars was significantly different. The DPPH free radical scavenging capacity of blackberry and hybrid cultivars was stronger in the fruiting period, decreased sharply, and plateaued in the final period (Figure 4). The free radical scavenging capacity of blackberry cultivars “Chester” and “Hull” showed the largest decline. The DPPH free radical scavenging capacity of each raspberry cultivar showed little difference in each period, but the scavenging capacity was also the highest in green fruit.

### 3.5. The Relationship between Fruit Biologically Active Substances and Antioxidant Capacity

Principal component analysis (PCA) showed that the first, second, and third principal components explained 46.74%, 17.96%, and 15.17% of the variance in the data, respectively (Table 2). The total contribution rate was 79.86%, so these three principal components sufficiently represented the original nine physiological indicators for analysis. The first principal component was negatively correlated with fructose, glucose, and soluble sugars and positively correlated with TFC, TPC, and DPPH free radical scavenging capacity (Table 3). The second principal component had a positive load on each index (Table 3). The third principal component was positively correlated with TAC (Table 3). In addition, the correlation analysis results showed that VEC, TFC, TPC, and DPPH free radical scavenging capacities were extremely significantly positively correlated (Table 4). TAC was significantly positively correlated with SSC and GC. TFC and DPPH free radical scavenging capacity were extremely significantly correlated (Table 4). There were slight differences in the correlation between bioactive substances and antioxidant capacity among different cultivars (Supplementary Table S4–S8). The DPPH free radical scavenging capacity of blackberry (“Chester” and “Hull”) was significantly positively correlated with VCC, VEC, TFC, and TPC (Supplementary Tables S2 and S3). The DPPH free radical scavenging capacity of the “Boysen” cultivar was significantly negatively correlated with TAC (Supplementary Table S4). The DPPH free radical scavenging capacity of the “Clode Summit” cultivar was significantly positively correlated with both VEC and TPC (Supplementary Table S6). These results indicated that VE, TF, and TP were closely related to the scavenging capacity of DPPH free radicals.

## 4. Discussion

This study subdivided different Rubus cultivars and fruit development stages and found that the fruits of blackberry and raspberry showed significant differences in fruit size during the growth and development process. The fruit size and weight of blackberry were significantly higher than those of raspberry. The fruit quality of black raspberry was significantly lower than that of blackberry or hybrids at all stages. This study concluded that the weight of “Heritage” fruits decreased after maturity, while Mikulic-Petkovsek et al. [25] weighed four types of blackberries during the ripening period and found that the weight of the fruit decreased near the end of the harvest season, similar to the results of this article. This observation may be due to the large amount of transpiration during the maturity period and the loss of water, which causes the weight of the berries to decrease. This study found that the TAC of yellow raspberry was extremely low, and the TAC of red raspberry was also significantly lower than that of black raspberry and blackberry, indicating that the darker the fruit color was, the greater the TAC may be, and revealing that the color change of Rubus fruit is closely related to flavonoids, including anthocyanins [26].

In terms of fruit flavor, sweetness is an important indicator for judging fruit quality. In this study, the sugar content of fruits of seven Rubus cultivars was determined, and it was found that the sugars in blackberry and raspberry cultivars were mainly glucose and fructose, which was consistent with the experimental results of Moraes et al. [12]. Compared with glucose, fructose has higher sweetness [27]. Kafkas et al. [28] tested the types and contents of five blackberry sugars, and the content of fructose was the most abundant sugar among all cultivars. This study found that, except for the levels of glucose and fructose in “Young” and “Hull” cultivars, the content of fructose in the other five cultivars was generally higher than that of glucose, similar to the results of Kefayati and Kafkas [29]. Yellow raspberry had the highest sugar content, with fructose accounting for 67.46%, which was the highest proportion of fructose among all seven cultivars, indicating that yellow raspberry had better palatability. The glucose content of “Young” fruits was highest when the fruits were about to ripen and slightly decreased when the fruits were fully ripe. This result may be due to sampling problems because overripe “Young” fruits consume glucose. Soluble protein may be used as an energy source for the synthesis of secondary metabolites. Therefore, the content of soluble protein was generally higher in the early stage of fruit development.

The antioxidant capacity of fruit mainly comes from active substances such as total phenols, flavonoids, and vitamins [30,31,32]. Kim et al. [33] analyzed the composition, content, and antioxidant activity of bioactive compounds in different cultivars of raspberry and blackberry and found that yellow raspberries contained no anthocyanins, but the TFC was significantly higher than that of other raspberry. The TAC was also extremely low in yellow raspberry, but the TFC was relatively higher than that in red raspberry. It has been confirmed that there is a positive correlation between the antioxidant capacity of raspberry and the TPC and TFC [34]. Correlation analysis of flavonoids and the antioxidant capacity of the fruits of seven Rubus cultivars showed that the abovementioned correlation was also true for blackberry. However, the TFC in blackberry in the first two growth periods was significantly higher than that in the other periods. Hybrids and raspberry only had an extremely high TFC in the fruiting period. The change in TFC in highbush blueberry is also the same, with the highest content in green fruits and the lowest content in mature fruits [35]. This result may be due to the conversion of total phenolics to anthocyanins during the fruit development stage and an overall decrease in the content of other phenolic components [36]. In the fruit development of jujube [37], the TFC gradually decreases with the expansion of the red area of the peel. The TPC was also the highest during the fruit development period. Duan et al. [38] believed that the synthesis of phenolic substances mainly occurs in the early stage of fruit development. Moreover, due to fruit expansion, the dilution of total phenols also reduces the relative content [39]. Given the high economic value and market popularity of blackberry and raspberry, we recommend that “Chester”, which had a higher yield, is suitable for planting from the perspective of growers; “Bristol” is suitable for extracting anthocyanins for the highest yield, and different cultivars can be selected for production or experimentation according to different needs.

This study found that the overall antioxidant capacity of the fruit was significantly positively correlated with the VEC, TFC, and TPC in the fruit. In most plants, the higher the TFC and TPC, the stronger the antioxidant capacity [40]. Vitamin E also has significant antioxidant capacity [41]. In Rubus fruits, these three types of substances likely synergistically regulate the antioxidant capacity of the fruit. In this study, we also found that vitamin E is abundant in the early stage of fruit development. The contribution rate of oxidizing power may be relatively high. The antioxidant capacity of blackberry (“Chester” and “Hull”) was also significantly positively correlated with VCC in the fruit, especially in the early stages of fruit development. The VCC of “Chester” and “Hull” fruits was significantly higher in the early stages of fruit development than that in other periods. This study found that the changes in the TFC in the seven Rubus cultivars were extremely significant or significantly positively correlated with those in the TPC, while the changes in the TAC in each cultivar were highly negatively correlated with those in the TFC. Anthocyanins are downstream products of flavonoids, and their synthetic pathways all start from the phenylalanine biosynthesis pathway. The genes encoding key enzymes of the phenylalanine metabolic pathway in blackberry fruits have the highest expression levels in green fruits, while the enzyme genes shared by the flavonol and anthocyanin synthesis pathways have the highest expression levels in mature fruits [42,43]. The biosynthesis of anthocyanins may compete for the same substrate, resulting in a highly negative correlation between the TAC and TFC. Related studies also showed that the high expression of some genes can significantly increase the production of phenolics and significantly increase antioxidant activity [44]. Moreover, the branches of the flavonoid synthesis pathway are also regulated by transcription factors (TFs). MYB can upregulate the synthesis of anthocyanins [45], and it can also inhibit the synthesis of anthocyanins in some species [46]; the same is true for flavonoids [47]. TFs may regulate the synthesis or accumulation of flavonoids and anthocyanins at the same time in blackberry. The specific molecular mechanisms regulating the synthesis of Rubus flavonoids and anthocyanins need to be further studied.

## 5. Conclusions

In conclusion, the accumulation of sugar was mainly in the later stages of Rubus fruit development and was dominated by fructose. The contents of total phenols and flavonoids were the highest in the green fruit period, and anthocyanins accumulated in the red and purple fruit periods. In seven cultivars, the weight of mature “Chester” fruits was the largest, “Clode Summit” fruits had the highest sugar content, and “Bristol” fruits had the highest TAC. The antioxidant capacity of blackberry and raspberry was generally positively correlated with the TPC. Flavonoids play a major role in antioxidant capacity and are also related to vitamins and other substances. This study provides a reference for the comparison of the content of the main active substances in different cultivars of Rubus and the correlations between the main nutrients.

## Figures and Tables

**Table 1:** Appearance index of fruits of different *Rubus* cultivars at different developmental stages.

Cultivar	Period	Horizontal Diameter (mm)	Longitudinal Diameter (mm)	Weight (g)	Hardness (kg·cm^−2^)
Chester	Green	10.82 ± 0.79 aB	11.65 ± 1.41 aC	0.95 ± 0.30 aC	
	Green–red	12.04 ± 0.84 aA	12.95 ± 1.83 aAB	1.00 ± 0.54 aA	
	Red	18.46 ± 0.83 bE	21.90 ± 1.58 bC	4.00 ± 0.71 bB	
	Red–purple	20.87 ± 0.93 cD	22.50 ± 1.19 bBC	4.79 ± 0.61 bB	0.44 ± 0.05 aC
	Purple	23.22 ± 1.67 dC	27.35 ± 1.41 cC	6.84 ± 1.20 cC	0.18 ± 0.05 bB
Hull	Green	11.89 ± 0.61 aC	15.50 ± 1.00 aE	1.35 ± 0.28 aD	
	Green–red	14.73 ± 0.80 bBC	17.91 ± 1.39 bC	2.04 ± 0.33 aC	
	Red	18.34 ± 1.51 cE	22.77 ± 2.82 cC	3.99 ± 0.94 bB	
	Red–purple	19.87 ± 1.28 dD	24.43 ± 2.19 cdC	4.92 ± 0.69 cC	0.39 ± 0.05 aCD
	Purple	19.99 ± 0.93 dB	25.50 ± 1.76 dBC	5.21 ± 0.83 cC	0.18 ± 0.06 bB
Boysen	Green	12.39 ± 0.61 aC	16.03 ± 0.66 aE	1.24 ± 0.16 aD	
	Green–red	15.41 ± 0.97 bC	19.12 ± 2.44 bC	2.30 ± 0.51 bC	
	Red	17.55 ± 1.41 cDE	20.53 ± 2.05 bcC	3.34 ± 0.67 cB	
	Red–purple	18.14 ± 0.87 cC	22.06 ± 1.56 cB	3.39 ± 0.64 cB	0.44 ± 0.10 aC
	Purple	19.85 ± 1.20 dB	24.98 ± 3.81 dBC	5.12 ± 1.22 dC	0.19 ± 0.09 bB
Young	Green	10.28 ± 0.55 aB	13.02 ± 0.85 aD	0.72 ± 0.11 aBC	
	Green–red	14.10 ± 0.87 bB	18.34 ± 1.23 bC	1.99 ± 0.16 bC	
	Red	16.16 ± 1.93 cCD	22.56 ± 1.77 cC	3.38 ± 0.61 cB	
	Red–purple	16.10 ± 1.20 cB	22.97 ± 2.89 cBC	3.40 ± 0.84 cB	0.33 ± 0.06 aABC
	Purple	18.73 ± 1.82 dB	21.37 ± 2.82 cB	3.55 ± 0.60 cB	0.10 ± 0.06 bA
Clode Summit	Green	9.06 ± 0.39 aA	8.45 ± 0.84 aA	0.40 ± 0.05 aA	
	Multicolor	12.97 ± 0.92 bA	13.18 ± 0.90 bAB	1.23 ± 0.18 bA	0.25 ± 0.09 aA
	Yellow	16.25 ± 1.51 cA	15.65 ± 1.00 cA	3.32 ± 0.35 cB	0.04 ± 0.02 bA
Heritage	Green	10.15 ± 0.79 aB	9.63 ± 0.74 aB	0.54 ± 0.08 aAB	
	Green–red	14.93 ± 1.12 bBC	13.58 ± 1.21 bB	1.44 ± 0.22 bB	
	Red	15.21 ± 0.72 bC	14.15 ± 2.65 bB	1.62 ± 0.58 bA	0.36 ± 0.06 aBCD
	Dark Red	15.59 ± 1.27 bB	12.90 ± 2.69 bA	1.46 ± 0.49 bA	0.21 ± 0.06 bB
Bristol	Green	9.10 ± 0.68 aA	8.24 ± 0.64 aA	0.38 ± 0.06 aA	
	Green–red	12.64 ± 0.75 bA	10.93 ± 0.99 bA	1.15 ± 0.18 bAB	
	Red	13.21 ± 1.06 bB	11.50 ± 1.11 bcA	1.25 ± 0.27 bA	
	Red–purple	13.62 ± 0.69 bA	12.52 ± 1.06 cA	1.25 ± 0.19 bA	1.62 ± 0.09 aAB
	Purple	15.32 ± 0.78 cA	14.08 ± 1.12 dA	1.88 ± 0.28 cA	1.46 ± 0.04 bA

Different lowercase letters (a–d) in columns denote significant differences between sampling dates for each cultivar by Duncan’s multiple range test (*p* < 0.05). Different capital letters (A–E) in columns denote significant differences by Duncan’s multiple range test (*p* < 0.05) among different cultivars.

**Table 2:** Eigenvalues and cumulative contribution rates of nine variances.

Component	Eigenvalues	Percent of Variance Explained/%	Cumulative Variance Contribution Rate/%
1	4.21	46.74	46.74
2	1.62	17.96	64.69
3	1.37	15.17	79.86
4	0.76	8.40	88.26
5	0.37	4.09	92.35
6	0.33	3.70	96.05
7	0.22	2.46	98.51
8	0.11	1.20	99.71
9	0.03	0.29	100.00

**Table 3:** Loading coefficients of each principal component.

	Component		
	1	2	3
Fructose	−0.698	0.41	−0.07
Glucose	−0.707	0.34	0.20
Soluble Sugar	−0.737	0.62	−0.07
Vitamin C	−0.028	0.54	−0.74
Vitamin E	0.702	0.35	0.46
Flavonoids	0.838	0.35	0.18
Anthocyanins	−0.421	0.36	0.70
Phenols	0.822	0.44	−0.03
DPPH free radical scavenging capacity	0.794	0.31	−0.18

**Table 4:** Correlation of active substances and antioxidant capacity.

	Fructose	Glucose	Soluble Sugar	Vitamin C	Vitamin E	Flavonoids	Anthocyanins	Phenols	DPPH
Fructose	1	0.35	0.84 **	0.13	−0.29	−0.48 **	0.28	−0.33	−0.47 **
Glucose		1	0.67 **	0.12	−0.33	−0.40 *	0.52 **	−0.47 **	−0.44 *
Soluble Sugar			1	0.32	−0.34	−0.37 *	0.41 *	−0.33	−0.38 *
Vitamin C				1	−0.17	−0.02	−0.18	0.21	0.26
Vitamin E					1	0.71 **	0.07	0.72 **	0.51 **
Flavonoids						1	−0.14	0.82 **	0.72 **
Anthocyanins							1	−0.24	−0.28
Phenols								1	0.68 **
DPPH									1

Note: “*”denotes significant differences between various indicators by Duncan’s multiple range test (*p* < 0.05). “**” denotes significant differences between various indicators by Duncan’s multiple range test (*p* < 0.01).

## Data Availability

The data and materials supporting the conclusions of this study are included within the article.

## Supplementary Materials

The following supporting information can be downloaded at: https://www.mdpi.com/article/10.3390/foods11081169/s1. Table S1. The protein content of different Rubus cultivars at different developmental stages; Table S2. Correlation of biologically active substances and antioxidant capacity in “Chester” fruits; Table S3. Correlation of biologically active substances and antioxidant capacity in “Hull” fruits; Table S4. Correlation of biologically active substances and antioxidant capacity in “Boysen” fruits; Table S5. Correlation of biologically active substances and antioxidant capacity in “Young” fruits; Table S6. Correlation of biologically active substances and antioxidant capacity in “Clode Summit” fruits; Table S7. Correlation of biologically active substances and antioxidant capacity in “Heritage” fruits; and Table S8. Correlation of biologically active substances and antioxidant capacity in “Bristol” fruits.
