# Supplementary material for: Variation in Bioactive Compounds and Antioxidant Activity of Rubus Fruits at Different Developmental Stages
Source: Foods. 2022 Apr 18;11(8):1169. doi: 10.3390/foods11081169 (PMC9026527; doi:10.3390/foods11081169)
Supplement: Supplementary file 1 [file foods-11-01169-s001.zip › foods-1679935-supplementary.pdf]

foods

Supplementary Materials

Table S1. The protein content of different *Rubus* cultivars at different developmental stages.

Cultivar	Period				
Chester	Green<br>3.73±0.51aB	Green-red<br>3.05±0.20bA	Red<br>1.33±0.13dB	Red-purple<br>1.39±0.22dA	Purple<br>2.06±0.20cA
Hull	Green<br>2.92±0.56aC	Green-red<br>0.95±0.20bcCD	Red<br>0.46±0.16cD	Red-purple<br>0.95±0.30bcB	Purple<br>1.34±0.36bB
Boysen	Green<br>2.36±0.31aCD	Green-red<br>0.75±0.20cD	Red<br>0.83±0.15cCD	Red-purple<br>1.06±0.06bcBC	Purple<br>1.30±0.28bB
Young	Green<br>1.43±0.12aE	Green-red<br>1.03±0.24bcBCD	Red<br>0.92±0.27bcC	Red-purple<br>1.24±0.06abBC	Purple<br>0.86±0.20cB
Clode Summit	Green<br>0.94±0.22bE	Multicolor<br>1.35±0.05bB	Yellow<br>1.91±0.37aA		
Heritage	Green<br>2.16±0.36aD	Green-red<br>1.36±0.11bB	Red<br>1.22±0.09bBC	Dark Red<br>1.29±0.16bBC	
Bristol	Green<br>4.70±0.32aA	Green-red<br>1.17±0.19bcBC	Red<br>0.84±0.17cCD	Red-purple<br>1.00±0.28bc	Purple<br>1.34±0.27bB

Note: Different lowercase letters (a–d) in columns denote significant differences between sampling dates for each cultivar fruits by Duncan's multiple range test (p<0.05). Different capital letters (A–E) in columns denote significant differences by Duncan's multiple range test (p<0.05) among different cultivars.

Table S2. Correlation of biologically active substances and antioxidant capacity in 'Chester' fruits.

	Fructose	Glucose	Soluble sugar	Vitamin C	Vitamin E	Flavonoids	Anthocyanins	Phenols	DPPH
Fructose	1								
Glucose	.976**	1							
Soluble sugar	.964**	.911**	1						
Vitamin C	-0.029	-0.186	-0.01	1					
Vitamin E	-.738*	-.840**	-.695*	.672*	1				
Flavonoids	-0.488	-0.627	-0.443	.873**	.935**	1			
Anthocyanins	.975**	.945**	.990**	-0.112	-.775**	-0.536	1		
Phenols	-0.354	-0.475	-0.377	.919**	.862**	.957**	-0.461	1	
DPPH	-0.502	-.633*	-0.48	.868**	.941**	.992**	-0.57	.973**	1

Note: "*" denotes significant differences between various indicators by Duncan's multiple range test (p<0.05). "**" denotes significant differences between various indicators by Duncan's multiple range test (p<0.01).

Table S3. Correlation of biologically active substances and antioxidant capacity in 'Hull' fruits.

	Fructose	Glucose	Soluble sugar	Vitamin C	Vitamin E	Flavonoids	Anthocyanins	Phenols	DPPH
Fructose	1								
Glucose	.997**	1							
Soluble sugar	.999**	.995**	1						
Vitamin C	-0.278	-0.286	-0.29	1					
Vitamin E	-0.071	-0.098	-0.069	.862**	1				
Flavonoids	-0.441	-0.471	-0.435	.778**	.900**	1			
Anthocyanins	.987**	.976**	.992**	-0.302	-0.044	-0.393	1		
Phenols	-0.473	-0.48	-0.481	.947**	.866**	.896**	-0.487	1	
DPPH	-0.499	-0.504	-0.508	.962**	.821**	.842**	-0.518	.989**	1

Note: "*" denotes significant differences between various indicators by Duncan's multiple range test ($p<0.05$). "**" denotes significant differences between various indicators by Duncan's multiple range test ($p<0.01$).

Table S4. Correlation of biologically active substances and antioxidant capacity in 'Boysen' fruits.

	Fructose	Glucose	Soluble sugar	Vitamin C	Vitamin E	Flavonoids	Anthocyanins	Phenols	DPPH
Fructose	1								
Glucose	.953**	1							
Soluble sugar	.938**	.866**	1						
Vitamin C	0.137	0.03	-0.002	1					
Vitamin E	-.731*	-.664*	-.891**	0.343	1				
Flavonoids	-0.391	-0.289	-.677*	0.384	.854**	1			
Anthocyanins	0.592	0.614	0.547	-0.53	-.664*	-0.306	1		
Phenols	-0.386	-0.3	-.676*	0.368	.853**	.993**	-0.265	1	
DPPH	-0.338	-0.223	-.637*	0.335	.811**	.995**	-0.22	.989**	1

Note: "*" denotes significant differences between various indicators by Duncan's multiple range test ($p<0.05$). "**" denotes significant differences between various indicators by Duncan's multiple range test ($p<0.01$).

Table S5. Correlation of biologically active substances and antioxidant capacity in 'Young' fruits.

	Fructose	Glucose	Soluble sugar	Vitamin C	Vitamin E	Flavonoids	Anthocyanins	Phenols	DPPH
Fructose	1								
Glucose	.991**	1							
Soluble sugar	.922**	.882**	1						
Vitamin C	-0.57	-0.616	-0.472	1					
Vitamin E	-0.475	-0.43	-.666*	0.547	1				
Flavonoids	-0.268	-0.222	-0.507	0.483	.970**	1			
Anthocyanins	.991**	.968**	.941**	-0.547	-0.554	-0.35	1		
Phenols	-.707*	-.640*	-.890**	0.338	.853**	.750*	-.777**	1	
DPPH	-0.58	-0.526	-.822**	0.455	.904**	.866**	-.636*	.919**	1

Note: "*" denotes significant differences between various indicators by Duncan's multiple range test ($p<0.05$). "**" denotes significant differences between various indicators by Duncan's multiple range test ($p<0.01$).

Table S6. Correlation of biologically active substances and antioxidant capacity in 'Clode Summit' fruits.

	Fructose	Glucose	Soluble sugar	Vitamin C	Vitamin E	Flavonoids	Anthocyanins	Phenols	DPPH
Fructose	1								
Glucose	.976**	1							
Soluble sugar	.977**	.981**	1						
Vitamin C	.988**	.983**	.975**	1					
Vitamin E	-0.725	-0.79	-0.803	-0.683	1				
Flavonoids	-0.357	-0.461	-0.491	-0.315	.899*	1			
Anthocyanins	.950**	.969**	.975**	.928**	-.902*	-0.627	1		
Phenols	-0.662	-0.735	-0.755	-0.62	.996**	.935**	-.861*	1	
DPPH	-.885*	-.914*	-.932**	-.851*	.949**	0.731	-.982**	.922**	1

Note: "*" denotes significant differences between various indicators by Duncan's multiple range test ($p<0.05$). "**" denotes significant differences between various indicators by Duncan's multiple range test ($p<0.01$).

Table S7. Correlation of biologically active substances and antioxidant capacity in 'Heritage' fruits.

	Fructose	Glucose	Soluble sugar	Vitamin C	Vitamin E	Flavonoids	Anthocyanins	Phenols	DPPH
Fructose	1								
Glucose	.975**	1							
Soluble sugar	.984**	.971**	1						
Vitamin C	-0.507	-0.554	-0.421	1					
Vitamin E	-.719*	-.771*	-0.643	.908**	1				
Flavonoids	-.725*	-.806*	-0.674	.795*	.960**	1			
Anthocyanins	.930**	.911**	.969**	-0.205	-0.453	-0.508	1		
Phenols	-.810*	-.875**	-.767*	.788*	.959**	.990**	-0.612	1	
DPPH	-.813*	-.875**	-.771*	.784*	.954**	.987**	-0.618	.999**	1

Note: "*" denotes significant differences between various indicators by Duncan's multiple range test ($p<0.05$). "**" denotes significant differences between various indicators by Duncan's multiple range test ($p<0.01$).

Table S8. Correlation of biologically active substances and antioxidant capacity in 'Bristol' fruits.

	Fructose	Glucose	Soluble sugar	Vitamin C	Vitamin E	Flavonoids	Anthocyanins	Phenols	DPPH
Fructose	1								
Glucose	.991**	1							
Soluble sugar	.979**	.957**	1						
Vitamin C	.707*	.676*	.821**	1					
Vitamin E	.678*	.732*	0.522	0.069	1				
Flavonoids	-0.368	-0.294	-0.52	-.712*	0.388	1			
Anthocyanins	.847**	.824**	.775**	0.302	.729*	-0.152	1		
Phenols	-0.339	-0.265	-0.481	-.636*	0.385	.993**	-0.172	1	
DPPH	-.857**	-.823**	-.934**	-.925**	-0.268	0.599	-0.565	0.529	1

Note: "*" denotes significant differences between various indicators by Duncan's multiple range test ($p<0.05$). "**" denotes significant differences between various indicators by Duncan's multiple range test ($p<0.01$).
